# A randomised controlled trial assessing the potential of palmitoylethanolamide (PEA) to act as an adjuvant to resistance training in healthy adults: a study protocol

**DOI:** 10.1186/s13063-023-07199-y

**Published:** 2023-03-31

**Authors:** Zoya Huschtscha, Jackson J. Fyfe, Simon A. Feros, Andrew C. Betik, Christopher S. Shaw, Luana C. Main, Gavin Abbott, Sze-Yen Tan, Martin C. Refalo, Michael Gerhardy, Emma Grunwald, Anthony May, Jessica Silver, Craig M. Smith, Matthew White, D. Lee Hamilton

**Affiliations:** 1 Institute for Physical Activity and Nutrition (IPAN), School of Exercise and Nutrition Sciences, Deakin University, Geelong, 3216 Australia; 2 Centre for Sport Research (CSR), School of Exercise and Nutrition Sciences, Deakin University, Geelong, 3216 Australia; 3 School of Exercise and Nutrition Sciences, Deakin University, Geelong, 3216 Australia; 4 Institute for Mental and Physical Health and Clinical Translation (IMPACT), School of Medicine, Deakin University, Geelong, 3216 Australia

**Keywords:** Palmitoylethanolamide, Pain, Hypertrophy, Strength, Inflammation

## Abstract

**Background:**

Non-steroidal anti-inflammatory drugs (NSAIDs) and analgesics are used frequently by athletes either prophylactically for the prevention of pain, or to accelerate recovery following an injury. However, these types of pain management strategies have been shown to inhibit signalling pathways (e.g., cyclooxygenase-2) that may hinder muscular adaptations such as hypertrophy and strength. Nutraceuticals such as palmitoylethanolamide (PEA) have analgesic properties that act via different mechanisms to NSAIDS/analgesics. Furthermore, PEA has been shown to have a positive effect on sleep and may contribute positively to muscle hypertrophy via PKB activation. Although PEA has not been widely studied in the athletic or recreationally active population, it may provide an alternative solution for pain management if it is found not to interfere with, or enhance training adaptations. Therefore, the study aim is to investigate the effects of daily PEA supplementation (Levagen + ®) with resistance training on lean body mass, strength, power and physical performance and outcomes of recovery (e.g., sleep) compared to placebo.

**Methods:**

This double-blind, randomised controlled study will take place over an 11-week period (including 8-weeks of progressive resistance training). Participants for this study will be 18–35 years old, healthy active adults that are not resistance trained. Participants will attend a familiarisation (week 0), pre-testing (week 1) and final-testing (week 11). At the pre-testing and final-testing weeks, total lean body mass (dual-energy X-ray absorptiometry; DXA), total mid-thigh cross sectional area (pQCT), maximal muscular strength (1 repetition maximum bench press, isometric mid-thigh pull) and power (countermovement jump and bench throw) will be assessed. Additionally, circulating inflammatory cytokines and anabolic hormones, sleep quality and quantity (ActiGraph), pain and subjective wellbeing (questionnaires) will also be examined.

**Discussion:**

This study is designed to investigate the effects that PEA may have on pre-to post intervention changes in total body and regional lean muscle mass, strength, power, sleep, subjective wellbeing, and pain associated with resistance training and menstruation compared with the placebo condition. Unlike other NSAIDs and analgesics, which may inhibit muscle protein synthesis and training adaptations, PEA which provides analgesia via alternative mechanisms may provide an alternative pain management solution. It is therefore important to determine if this analgesic compound interferes with or enhances training adaptations so that athletes and active individuals can make an informed decision on their pain management strategies.

**Trial registration:**

Australian New Zealand Clinical Trials Registry (ANZCTR: ACTRN12621001726842p).

**Supplementary Information:**

The online version contains supplementary material available at 10.1186/s13063-023-07199-y.

## Background and rationale

Body composition and power-to-weight ratio are acknowledged as key performance determinants in a variety of different sporting modalities [1]. Both variables rely on athletes possessing sufficient skeletal muscle mass (relative to fat mass) and muscle quality (e.g., strength/power relative to muscle mass), and these outcomes differ between sub-elite and elite athletes in a range of different sports [2–5]. For example, lower body fat and higher power-to-weight ratio are important for sports that rely on power, speed, and agility such as martial arts, boxing, gymnastics, and team sports such as soccer and rugby [6, 7]. Additionally, increasing muscle mass is a common pursuit for many recreationally active individuals who aim to optimise their body composition and strength for aesthetic reasons or long-term health (e.g., healthy ageing and metabolic risk factors) [8, 9]. Acknowledging that skeletal muscle mass, strength, and power are key elements in any athletic development program, understanding methods to optimise these outcomes while minimising increases in body fat is of great value to many athletes as well as the general population.

The management of pain arising either from injury (e.g., acute or overuse) or factors associated with exercise and training [such as delayed onset muscle soreness (DOMS)] is important for athletes as it can influence immediate and future performance. Pain management is often overlooked in athletic preparation and athletes that experience pain may lose training time or withdraw from competition altogether [10]. Furthermore, female athletes who experience pain prior to- or during the menstrual phase of their cycle have reported that it negatively impacts their performance during training or competition [11]. Lastly, pain can also affect sleep quality in a reciprocal relationship [12, 13]. For example, athletes that report inadequate sleep time or quality (e.g., restful sleep vs restless) are at increased risk of injury, illness susceptibility, weight gain and decreased pain tolerance [13]. Consequently, athletes report frequently ingesting non-steroidal anti-inflammatory drugs (NSAIDs) prophylactically for the prevention of pain, or to speed recovery following an injury [14–16]. One study reported that elite Olympic level athletes are 3.6 times more likely to use NSAIDs than age-matched controls [17]. Additionally, reports in elite level football players have reported 10–20% of athletes taking NSAIDs either prior to almost every match [18]. Many common pain management agents, such as NSAIDs, ibuprofen, and analgesic paracetamol, reduce pain by inhibiting cyclooxygenase-2 (COX-2) [19–22]. Inhibition of COX-2 negatively affects the anabolic signalling pathways that induce the muscle protein synthetic response to acute exercise, potentially impairing long-term physiological adaptations [19–22]. Therefore, it may be beneficial to explore alternatives to pain management for athletes that do not have the potential negative side effects associated with COX-2 inhibitors.

Nutraceuticals are dietary supplements derived from food sources that have added benefits beyond those obtained from food sources alone [23]. In preliminary studies, nutraceuticals are showing promising benefits in augmenting adaptations in skeletal muscle mass following resistance training either directly or indirectly but are not widely studied in the literature [23, 24]. Palmitoylethanolamide (PEA) is a nutraceutical compound that has supported analgesic effects for both acute and chronic pain [25, 26]. PEA doses of between 300 -1200 mg have been well-tolerated and shown to be effective at reducing pain associated with osteoarthritis, and it can reduce markers of skeletal muscle damage following damaging exercise [26–28]. PEA has both anti-inflammatory and analgesic effects through a variety of receptor sites both directly [e.g., proliferator-activated receptor alpha (PPARα); G protein-coupled receptor 55 (GPR55)] and indirectly [e.g., Cannabinoid receptor type 1 and 2 (CB_1 and_ CB_2_); transient receptor potential cation channel subfamily V member 1 (TRVP1)] [29]. Unlike NSAIDs, ibuprofen, and analgesic paracetamol, which inhibit muscle protein synthesis [30], orally-dosed PEA increases protein kinase B (PKB) phosphorylation following exercise, which plays an integral role in the anabolic pathways promoting skeletal muscle hypertrophy [31]. Additionally, PEA may improve sleep quality and duration, which could further assist athletes’ recovery and therefore will likely be beneficial for athletes [32]. Unlike the negative side effects found in COX-2 inhibitors, the analgesic and anti-inflammatory properties of PEA could provide an alternative solution to pain management as well as contribute positively to muscle hypertrophy and sleep, all of which are likely to be highly beneficial for athletes and active individuals.

### Objectives

The primary aim of this study is to investigate the effects of daily PEA supplementation (Levagen + ®) during an 8-week period of resistance training on pre- to post-intervention changes in total body and regional lean body mass (e.g., mid-thigh) compared with a placebo condition. Our secondary aims are to explore the effects of PEA supplementation on changes in strength, power, sleep, subjective wellbeing, and pain associated with resistance training and menstruation.

### Trial design

This double-blind, randomised controlled study will take place over an 11-week period (including 8-weeks of progressive resistance training). Participants for this study will be 18–35 years old, healthy active adults that are not resistance trained. It is hypothesised that PEA supplementation together with resistance training will further improve muscle mass, strength and power, enhance wellbeing, reduce post exercise muscle soreness and menstrual pains, and improve sleep quality and this effect will be greater than the placebo group.

## Methods: Participants, interventions and outcomes

### Study setting

This study will take place at Deakin University, Burwood campus. This protocol is reported in accordance with the Standard Protocol Items: Recommendations for Intervention Trials (SPIRIT) [33]. Recruitment commenced in March 2022 and is expected to finish in March 2023. This study design, as well as all consent forms and research tools have been approved by Deakin University Human Research Ethics Committee (DUHREC: 2021–312).

### Eligibility criteria

Participants in this study will be males and females between the ages of 18–35 years old who will be recruited via online advertisements (e.g., Facebook) and study posters advertised around the local area. In response to the advertisement materials, interested individuals can: 1) directly email the study coordinator, or; 2) complete an online form which will provide contact details so that research staff can contact them directly. All interested individuals will be screened via telephone to determine eligibility. Participants will be eligible for inclusion into the study if they meet the following criteria:

### Inclusion criteria

- Recreationally active (a minimum of 150 min of self-reported moderate-intensity physical activity per week)
- Stable body mass for the last 2 months
- BMI between ≥ 18.5 or ≤ 28 kg/m.^2^

### Exclusion criteria

- Major musculoskeletal injury in the past 6 months
- Participation in regular structured resistance training in the past 6 months (e.g., two or more sessions per week)
- Allergies to any of the contents of the Levagen + ® supplement or placebo formulation
- Currently participating in a weight loss program or special diet (e.g., Low carbohydrate, ketogenic, vegan etc.)
- Smokers
- Current functional impairment that would limit participation in the intervention
- Use of sports supplements (e.g., creatine or protein powder) or pain medication in the last month
- Current chronic disease including: cancer, diabetes, cardiovascular disease, chronic liver disease, and gastrointestinal disorders that affect nutrient absorption
- Cognitive impairment or inability to commit to the study and its requirements

Once participants agree to participate, one of the researchers responsible for the study coordination (ZH) will provide the participant with a written plain language explanatory statement as well as verbally describe the study details. Participants will be provided both verbal and written information regarding the following: the purpose of the study, the study aims, the procedures of the study and what will be required from the participant during the 11-weeks, possible benefits and risks for participating, how their data will remain confidential and contact details for any problems concerning the project. During the familiarisation visit, prior to any testing, written consent will be obtained [appendix 1]. Participants will be informed that they are free to withdraw from the study and withdraw their consent at any time during the study [appendix 2]. To further promote study retention, participants will receive a $200 gift card upon completion of the study. During the study, participants will be asked to resume their usual physical activity and dietary habits and be asked to avoid taking any pain medication during the course of the study.

### Interventions

#### Supplements

Eligible participants will be asked to maintain their usual dietary intake and current exercise habits, abstain from using any pain medication, and sports supplements that are not provided during the course of the study. The intervention groups will be provided capsules of PEA (350 mg/day as 2 × 175 mg Levagen + ® capsules), or identical matching placebo capsules (in colour and weight) containing maltodextrin (2 × 175 mg capsules), which will be taken orally (swallowed) for 8-weeks. On training days, participants will be instructed to take 1 × capsule 45 - 60 min prior to exercise with their standardised meal (388 kcals, 45 g CHO, 21 g PRO, 13 g FAT) and 1 × capsule in the evening 60 min prior to sleep [32]. The supplement taken prior to exercise is provided based on the time to peak blood concentrations of PEA (~ 45–60 min) [34]. On non-training days, participants will be asked to consume 1 × capsule with their morning meal and 1 × capsule pre-sleep, 60-min prior as per training days. The dosage of the supplement within this study is well below previous studies that have shown dosages of up to 1,200 mg/day have no harmful effects [35]. In the rare case that a participant may experience undesirable side effects, the blind will be broken, and participants will be referred to a local general practitioner and they may withdraw from the study.

The trial products will be supplied by Gencor Pacific (Lantau Island, Hong Kong) and manufactured by Pharmako Biotechnologies (Sydney, Australia). Compliance with dosing at home will be assessed by pill count and a tracker sheet at the end of the study.

#### Resistance training

Participants will complete 8-weeks of supervised progressive resistance training twice per week on non-consecutive days at Deakin University Burwood campus. The sessions will be supervised by a qualified exercise trainer (e.g., exercise physiologist or qualified strength and conditioning coach) to monitor correct lifting and rest intervals. Each resistance training session (45 to 60 min in duration) will consist of eight exercises completed as four complex pairs. The exercises and complex pairs include: 1A leg press + 1B countermovement jump, 2A bench press + 2B bench press throw, 3A deadlift + 3B deadlift jumps, and 4A bench pull + 4B power bench pull. The strength-focused exercises (denoted with letter “A” of each complex pair) will be performed with a slow and controlled eccentric phase of ~ 2–3 s for increasing time under tension (stimulus for hypertrophy) and an explosive concentric phase (stimulus for strength and power development) with higher loads. The power-focused exercises (denoted with letter “B” of each complex pair) will be performed explosively (I.e., as fast as possible) in the eccentric and concentric phases with lower loads. The loads prescribed for the strength- and power-focused exercises will be calculated as percentages of the 1-RM either assessed directly (bench press) or predicted from a 6-RM (leg press, bench pull and deadlift) as determined in the familiarisation week**.** During weeks 3–8, an extra set will be performed for each of the strength-focused exercises whereby the load will be decreased (to 50 - 60% 1-RM) and participants will perform as many reps as possible (AMRAP). Some additional accessory exercises (e.g., leg curls, biceps curls and triceps pushdowns) to achieve muscle balance will also be added at this time. Rest between sets and exercises will range from 3–5 min. The prescription of the repetitions, sets and loads are outlined in Table 1.

**Table 1:** Repetition, sets and loads to be used during the 8-week progressive resistance training program

	**Sets**	**Repetitions**	**Load** **(Strength exercises)**	**Load** **(Power exercises)**
Weeks 1–2	3	6	75% 1RM	45% 1RM
Weeks 3–4	3	5 AMRAP	80% 1RM 50-60%	50% 1RM
Weeks 5–6	4	4 AMRAP	85% 1RM 50-60%	55% 1RM
Weeks 7–8	5	3 AMRAP	90% 1RM 50-60%	60% 1RM

*AMRAP* as many reps as possible

Following each resistance training session, participants will be provided with a flavoured whey protein concentrate beverage (250 Cals; 40 g protein) mixed with 200–300 ml of water to be consumed within 15 min of completing the session to support and maximise muscle protein synthesis and promote muscle hypertrophy adaptations.

#### Study outcomes

**Fig. 1:**
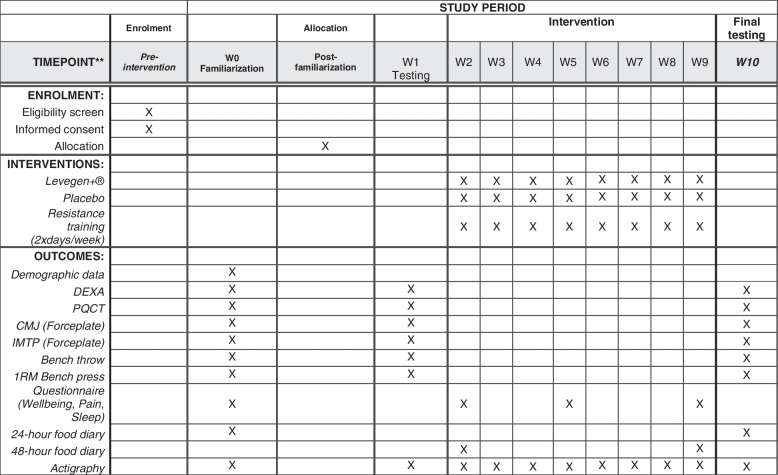
Schedule of enrolment, interventions and assessments

For all testing visits, participants will be required to arrive in a fasted state and to avoid any strenuous exercise in the 24-h prior to testing. The schedule of enrolment, interventions and assessments  is outlined in Fig. 1.

### Primary outcomes

#### Lean body mass

The primary outcomes will be the mean (± standard deviation, SD) difference between the two groups for total body (%) (DXA; Lunar Corp., Madison, WI) and regional (e.g., mid-thigh) lean muscle mass (%). Regional muscle mass will be assessed by the measurement of the mid-thigh cross sectional area (cm^2^) which will be measured by a Perpheral Quantitative Computered Tomography (pQCT;XCT 3000, Stratec Medizintechnik GmbH, Pforzheim, Germany). A tomographic slice will be taken 66% proximal from the distal tibia on the right leg, which is considered the largest cross-sectional area of the lower leg [36]. The pQCT results will be analysed using the BoneJ plugin for ImageJ [37]. Measurements for the DXA and pQCT will be taken at familiarisation (week 0), pre-testing (week 1) and post-testing (week 11) and all scans will be carried out by the same researcher (ZH). The outcomes taken over the two consecutive weeks (e.g., familiarisation and baseline) will allow for researchers to report the coefficient of variation (CV) values to report test-retest reliability. For both the pre-testing and post-testing visits participants will be asked to consume 500 ml of water 45–60 min prior to body composition testing to control for hydration status and to minimise the influence hydration status on DXA- derived measure. Confirmation of hydration status will also be carried out via measuring plasma osmolality (Osmomat 3000, Gonotec, Utah, United States of America) from plasma taken from venous blood samples in duplicate.

### Secondary outcomes

#### Blood collection and analysis

Following the primary outcome measures, while the participants remain fasted, a venous blood sample will be collected from the antecubital vein at pre-testing and at post-testing. Heparin whole blood samples will be centrifuged at 4,000 rpm (1,500 × g) for 10 min within 15 min of sample collection. Aliquots of heparin plasma will be placed in 1.5 ml microstorage tubes and frozen at − 80 °C until all samples have been collected. Once the study has concluded all biomarkers will be analysed. Anabolic biomarkers (e.g., testosterone and oestradiol) will be analysed by Enzyme-linked immunosorbent assay (ELISA) kits. Plasma concentrations of pro- and anti-inflammatory cytokines (e.g., Interleukin- -2, 6, 8, 10 etc.) will be analysed by a multiplex ELISA kit. Once all markers have been analysed extra samples will be discarded appropriately. Biochemistry markers will be reported as the mean difference between the groups using the specific and relevant metrics for each marker.

#### Muscle strength and power

Prior to any exercise testing participants will be provided with a standardised breakfast (388 kcals, 45 g CHO, 21 g PRO, 13 g FAT).

A force plate (400 s + Performance Force Plate, Fitness Technology, Adelaide, Australia) will be used to assess lower body strength and power during isometric mid-thigh pull (IMTP) and countermovement jump (CMJ) tests, respectively. The force plate will be interfaced with computer software (Ballistic Measurement System; Fitness Technology, Adelaide, Australia). The CMJ outcomes will be jump height (cm), peak force (N), peak power (W). The outcomes for the IMTP will be peak force (N) and peak power (W). Participants will undergo a standardised warm-up prior to performing 3 maximum-effort attempts of the CMJ and IMTP, respectively. The highest value of the 3-maximium efforts for the exercise will be analysed and reported to compare group differences.

Upper body power will be assessed using a bench throw performed using a Smith machine squat rack (Maxrack, IP-L8505, Star trac, Irvine, California, United States). A linear position transducer (GymAware v2.4.1, GymAware, Kinetic Performance Technology, Canberra, Australia) will be attached to the Smith machine barbell and used to assess barbell velocity (m/s) and power (W). Following a standardised warm-up, participants will perform 3 sets of 4 repetitions with a 2-min rest interval between sets. The highest value for the 3-maximum attempts for peak velocity (m/s) and peak power (W) will be recorded for each participant.

Lastly, upper body strength will be assessed by a 1-repetition maximum (1-RM, kg) bench press using a free weight bench press. Participants will first perform a warm-up involving 8, 5, and 3 repetitions with 50%, 75%, and 95% of predicted 1-RM, respectively. Participants then will perform 1-RM attempts with increasing loads (5–10 kg for males; 2.5–5 kg for females), with a 2.5 kg minimum load, until the 1-RM is reached. Participants will rest for 3 min in-between sets [38].

For all strength and power performance outcomes, the mean group difference at pre-testing vs post-testing will be reported.

#### Self-reported questionnaires

Questionnaire data will be collected using Research Electronic Data Capture (REDCap) via participants own phones using a QR code. All questionnaire data will be recorded at familiarisation (week 0) and in weeks 2, 5 and 9. Outcomes measured in the self-reported questionnaire includes measurements or pain, menstrual pain, overall wellbeing, and sleep all of which are detailed below:

- To measure perception of pain associated with DOMs, participants will be asked to ‘rank their current level of pain’ using a 10-point visual analogue scale.
- Only female participants will be required to fill in a premenstrual symptoms screening tool (PSST) [39]. Alongside the questionnaire female participants will also be asked to track their menstrual cycle during the study on an electronic calendar and will be asked to mark the days of their menstruation 1-month prior to commencing the trial as well as during. For females that are on or taking hormonal contraceptive methods, the type of contraceptive will be recorded.
- To measure subjective wellbeing, the Short Recovery and Stress Scale (SRSS) questionnaire will be used to monitor wellbeing [40].
- Sleep quality will be assessed using the Pittsburgh Sleep Quality Index (PSQI) [41].

### Actigraphy

Sleep and daily physical activity will be monitored continuously using an ActiGraph (ActiGraph GT9X, ActiGraph, Pensacola, FL) which will be worn by participants on their non-dominant wrist for the study period. Participants will be instructed to wear the activity monitor during the whole period of intervention trial including during sleep and to take the monitor off only when engaging in aquatic activities, or when the watch requires charging. The ActiGraph will be provided to participants on their familiarisation study testing day, which will allow for 1 week of baseline data collection.

### Food diary

To standardise food intake prior to testing, participants will be asked to complete a 24-h food and fluid diary the day before their pre-test session and asked to ingest the same food and fluids again the day before the post-test day of the study. Additionally, participants will also be asked to complete a 2-day (1 weekday and 1 weekend) food diary at the end of week 2 and week 9. This will be used to assess if major changes in their diet have occurred during the training period within groups and to report any between group differences.

### Sample size

It has been calculated that a sample size of 42 participants (21 per group) will provide 80% power, for α = 0.05, to detect a 2% difference between groups in percentage lean mass change [assumed SD = 2.2% based on data from [42] to detect a significant change in lean muscle mass. To allow for an ~ 20% dropout rate, we will aim to recruit 52 participants in total. As proof of principle, a study by Lilja et al. [19] demonstrated a significant difference in muscle mass and strength between participants treated with NSAIDs vs placebo after 8 weeks of resistance training with a participant pool of 35 participants. Sample size calculations were performed in Stata/SE 16.1 (StataCorp, TX) using the *power two means* command.

## Methods: Assignment of interventions (for controlled trials)

### Sequence generation

Allocations will be generated via stratified block randomisation, with blocks sizes of two and stratification factors being biological sex and appendicular skeletal muscle mass index (SMI kg/cm^2^: Male, Low SMI (≤ 8.09 kg/cm^2^:); Male, high SMI: (> 8.09 kg/cm^2^:); Female, low SMI (≤ 6.64 kg/cm^2^:); female high SMI (> 6.64 kg/cm^2^:) as measured by DXA. These values were determined based on the average SMI measured within the cohort. The data for the allocations will be collected by the researcher conducting the scans at the familiarisation testing day and submitted into an excel sheet to the researcher that is responsible for the group allocations. The randomisation will be carried out using a computer-generated randomisation table in Microsoft Excel. The randomisation allocations were produced with Sata module *ralloc*.

### Allocation concealment and blinding

The supplement and placebo are provided by Gencor Pacific Ltd and will be provided in white opaque identical containers labelled either ‘A’ or ‘B’. The contents of each container will be provided in a sealed envelope that will only be made available to the researcher responsible for the sequence allocation who is not associated with the study. The researchers responsible for data collection and participant management will both be blinded to the groups and will not be made aware of the group allocation until after the data analysis.

### Methods: data collection, management and, analysis

#### Data management and monitoring

Research assistants that are blinded to the supplement allocation will be trained on how to collect data. Data for body composition and exercise testing will be collected at the same location throughout the study. These data will be collected electronically, and research assistants will also backup electronic data using de-identified, participant coded data collection sheets. The electronic data is stored on password protected computers and the data sheets are stored in a locked filing cabinet. All research data collected will be transferred to an excel worksheet on password protected servers. While data is being collected, all data will be checked for omissions or errors by the project manager who will maintain a data diary of any issues that arise with the data. The data then will be checked again by the principal investigator (PI) and statistician prior to any analysis. The information collected from this study will be kept until the end of the project and then placed in archives for a minimum of 5 years from the final publication of the findings.

The CMJ, IMTP and bench throw will be taken in triplicate. A fourth measure may be taken if two of the measurements are not within ~ 10% of each other. Participants will have regular contact (2xdays/week) with the researchers and receive check-ins if they have missed sessions. Participants will have the opportunity to re-schedule missed sessions during the same week of training.

Any adverse effects recorded by participants will be reported directly to the PI. The PI will directly report adverse effects to the Therapeutic Goods Administration (TGA) in the Department of Health and Age Care, Australia. In the unlikely event of severe adverse reactions, the PI will make the final decision to terminate the trial.

#### Statistical methods

A primary analysis of intention to treat (ITT) will be conducted of all the participants that have completed the trial. A secondary, per-protocol analysis will be carried out to include only participants that attend ≥ 80% of their resistance training and consumed ≥ 80% of their supplement over the course of the study. Between-group comparisons will be made using linear models adjusting for baseline outcome level and stratification factors sex and SMI (low/high). Outcome variables will be log-transformed where necessary to satisfy parametric assumptions. All tests will be two-tailed with statistical significance set at p < 0.05. Multiple imputation by chained equations will be used to handle missing data.

#### Ancillary and post-trial care

The research team in this study aim to mitigate risk associated with the intervention, however potential small harms may occur. In the case that a participant is harmed during this research project, there are no special compensation agreements that have been made. However, in the event of a harm the participants will be encouraged and directed to contact the Deakin Human Research Ethics Committee.

## Discussion

For athletes, pain management either from injury or associated with training and exercise can influence athletes' immediate or future performances. Athletes have unrestricted access to over-the-counter pain management such as NSAIDs and other analgesic medications, and reports have indicated prolonged prophylactic use by some athletes [14–19]. Athletes that regularly take NSAIDs and other analgesics medications may be at risk of lower muscular adaptations due to the inhibitory effects these medications may have on intramuscular pathways such as the cyclooxygenase-2 (COX-2) [19–22]. The aim of this study is to explore an alternative option to pain management for athletes and to investigate the effects of daily PEA supplementation (Levagen + ®) on pre- to post-intervention changes in total body and regional lean body mass and mid-thigh cross-sectional area compared with a placebo condition during an 8-week period of resistance training. Our secondary aims are to explore the effects of PEA supplementation on changes in strength, power, sleep, subjective wellbeing, and pain associated with resistance training and menstruation. This study is the first of its kind to evaluate the effectiveness of PEA supplementation in the context of skeletal muscle hypertrophy to be applied to an active population.

The results of this study may assist active individuals, athletes and their coaches in making an informed decision about the most appropriate pain management strategy for them. If PEA is found not interfere with, or to enhance training induced muscle hypertrophy and improve sleep, then it may be a better pain relief option than OTC medications. These findings may be beneficial for those that work with and manage athletes such as sports physicians, coaches, strength and conditioning coaches and the athletes themselves. The findings from these studies will be presented at meetings, conferences and published in peer-reviewed scientific journals.

A potential limitation of this study is the exclusion of participants with experience in structured resistance training in the past 12-months. This may limit the generalisation of our findings to non-strength trained populations.

### Trial status

Recruitment began on the 21^st^ of March 2022. Recruitment is still ongoing and is due to be completed by March 2023. The study has been running according to protocol #4 and will continue to run according to this protocol until completion.

## Authorship

Authorship will be granted to those that fulfill The International Committee of Medical Journal Editors (ICMJE) four main criteria [43]. There is no intention to use professional writers.

## Supplementary Information

**Additional file 1.**Plain language statement and consent form.

**Additional file 2.**Withdrawal of consent form.

## Data Availability

The datasets used and/or analysed during the current study are available from the corresponding author on reasonable request.

## Abbreviations

1-RM: 1- Repetition maximum
AMRAP: As many reps as possible
BMI: Body mass index
Cals: Calories
CB_1_: Cannabinoid receptor type 1
CB_2_: Cannabinoid receptor type 1
COX-2: Cyclooxygenase-2
DOMS: Delayed onset muscle soreness
DXA: DXA- dual-energy X-ray absorptiometry
GPR55: G protein-coupled receptor 55
NSAIDs: Non-steroidal anti-inflammatory drugs
PEA: Palmitoylethanolamide
pQCT: Peripheral Quantitative Computed Tomography
PRO: Protein
PPARα: Proliferator-activated receptor alpha
SPIRIT- Standard Protocol Items: Recommendations for Intervention Trials
TRVP1: Transient receptor potential cation channel subfamily V member 1
