# Supplementary figures and images for: A randomised controlled trial assessing the potential of palmitoylethanolamide (PEA) to act as an adjuvant to resistance training in healthy adults: a study protocol
Source: Trials. 2023 Mar 31;24:245. doi: 10.1186/s13063-023-07199-y (PMC10064518; doi:10.1186/s13063-023-07199-y)

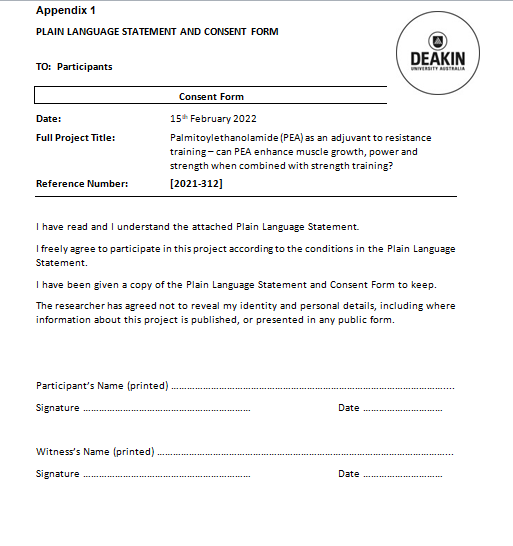

Supplement: Supplementary file 1 — Additional file 1.Plain language statement and consent form. [file 13063_2023_7199_MOESM1_ESM.png]

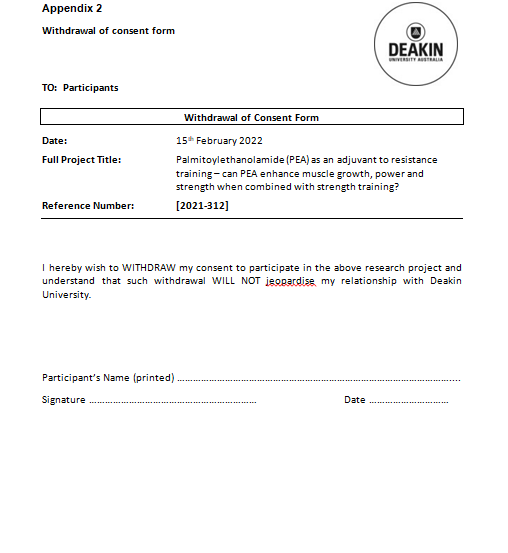

Supplement: Supplementary file 2 — Additional file 2.Withdrawal of consent form. [file 13063_2023_7199_MOESM2_ESM.png]
